# A Mosquito Workshop and Community Intervention: A Pilot Education Campaign to Identify Risk Factors Associated with Container Mosquitoes in San Pedro Sula, Honduras

**DOI:** 10.3390/ijerph16132399

**Published:** 2019-07-06

**Authors:** Casey Parker, Felicita Garcia, Oscar Menocal, Dunia Jeer, Barry Alto

**Affiliations:** 1 Florida Medical Entomology Laboratory, University of Florida, 200 9th St SE, Vero Beach, FL 32962, USA; 2 Department of Nursing, Universidad Nacional Autónoma de Honduras—Valle de Sula, 21102 San Pedro Sula, Honduras; 3 Department of Biology, Universidad Nacional Autónoma de Honduras—Valle de Sula, 21102 San Pedro Sula, Honduras

**Keywords:** *Aedes aegypti*, *Aedes albopictus*, community education, container mosquitoes, dengue, mosquito control, vector control

## Abstract

Dengue poses a significant public health threat and results in ~96 million clinical cases every year. Central America is a region burdened by neglected tropical diseases, including dengue. The primary vectors of dengue, *Aedes aegypti* and *Aedes albopictus*, are widely distributed in Honduras. Additionally, sustained and consistent mosquito control is lacking in the country. Successful control of container mosquitoes relies heavily on participation from community leaders, stakeholders, and the community itself. We conducted a pilot study in San Pedro Sula, Honduras where community leaders and stakeholders were trained on mosquito biology and control and were able to apply that knowledge to an underserved community in San Pedro Sula. Surveys to assess the number and type of containers in the community and the number of containers on the residence identified associations with select socioeconomic factors and other variables based on survey questions. The average number of containers on the premises was 15 (± 2.3) and the most prevalent containers (>50%) were flowerpots, garbage, and toys, which could be targeted in mosquito control programs. This pilot study offers a framework for training community leaders and stakeholders to create a sustainable community-based vector control program for container mosquitoes.

## 1. Introduction

Central America is burdened by neglected tropical diseases (NTDs), including dengue [1]. The association of NTD prevalence and poverty is well-established [2] and in Honduras, 62% of the population is living below the poverty line [3] and 17% live in extreme poverty (less than $1.90 per day) [4]. Honduras experiences millions of cases of NTDs annually and many more individuals are at risk of infection [1].

Dengue virus (DENV) *Flavivirus* (Flaviviridae) is considered a neglected tropical disease and is responsible for an estimated 390 million dengue infections per year (96 million clinical cases) [5]. Dengue fever is characterized by non-specific flu-like symptoms including headache, fever, nausea, vomiting, and rashes. However, more severe manifestations of the disease include dengue hemorrhagic fever and dengue shock syndrome, both of which are potentially fatal [6]. The global presence and severity of this disease makes it a notable public health concern.

Dengue is endemic in Honduras with close to one million cases every year [1]. Epidemics occur periodically as well and a recent epidemic in San Pedro Sula has already resulted in 12 deaths (June) in 2019 [7]. Honduras has faced an increase in public health challenges in recent years from other emerging mosquito-borne arboviruses including chikungunya (CHIKV) [8] and Zika virus (ZIKV) [9]. Symptoms of infection with these viruses are similar to dengue fever, but infection with ZIKV while pregnant can result in fetal microcephaly. Human illness from CHIKV is self-limiting [10], although chronic musculoskeletal diseases may persist for months to years after initial infection [11].

*Aedes aegypti* (Linnaeus) and *Aedes albopictus* (Skuse) are the primary vectors of DENV, CHIKV, and ZIKV [12,13,14] and both species are widely dispersed in Honduras [15]. Both of these mosquito species share similar biological and behavioral characteristics that make them prominent vectors of concern for human pathogens. *Aedes aegypti* and *Ae. albopictus* are considered container mosquitoes because they will oviposit in a variety of natural and artificial containers [16,17], including catch basins of storm drains, used tires, domestic containers, vases, phytotelmata, and cryptic container habitats. Often these containers are small and difficult to locate, making control using conventional methods problematic. The propensity to exploit human made containers coincides with proximity to human habitation. Both species exhibit “skip” oviposition, where females visit multiple sites while laying their eggs [18,19]. *Aedes aegypti* also preferentially takes bloodmeals from human hosts [20] and feeds primarily during the day when humans are most active [21]. Finally, *Ae. aegypti* will often feed more than once during a gonotrophic cycle, which increases vectorial capacity [22]. Vectorial capacity is an index and provides a measurement of risk of pathogen transmission. This combination of factors makes both *Ae. aegypti* and *Ae. albopictus* species of concern from a public health and control perspective.

The World Health Organization (WHO) states that “sustained mosquito control efforts are important to prevent outbreaks of emergent mosquito-borne diseases” [23], but in Honduras, mosquito control is usually carried out by the Ministry of Health in response to a disease epidemic [7]. Even when control measures are implemented, control of container mosquitoes can be difficult due to the cryptic and numerous types of larval habitats utilized and their daytime feeding behavior because they are missed with traditional night time adulticide treatments.

This lack of consistent and sustained mosquito control highlights the need for alternative control strategies for container mosquitoes in the region. Despite challenges, effective control of container mosquitoes can be achieved through sustainable community-based programs that target larval development sites [24,25]. A study conducted in El Progreso, Honduras launched an *Ae. aegypti* control program within the community and observed a reduction in larval indices as well as an increase in knowledge regarding dengue transmission and prevention among community members [26]. Similar community-based campaigns have been successful in other regions as well [27,28,29]. However, the importance of integrating community leaders and other stakeholders in efforts to eliminate container mosquito larval habitats is obviously critical to the success and sustainability of a community-based intervention program [30,31].

Eliminating the larval habitats where container mosquitoes develop can decrease the adult population of mosquitoes, and therefore influence the incidence of vector-borne disease in the area. In San Pedro Sula, Honduras, the prevalence of vector-borne diseases transmitted by container mosquitoes highlights the need for a community-based program in the area. Here, we describe a community-based campaign that trained community leaders and stakeholders on mosquito biology and control of container mosquitoes and then these individuals had the opportunity to work directly in a community where the workshop participants provided educational material to the community and shared knowledge and mosquito prevention strategies. During these community visits, surveys were used to investigate the association of socioeconomic factors with container prevalence and to determine the most prevalent container type within this community.

## 2. Materials and Methods

### 2.1. Study Site

San Pedro Sula, Honduras has a tropical climate with average temperatures ranging from 24–28 °C and annual precipitation of approximately 1400 mm. This climate is ideal for the development of container mosquitoes and, therefore, it was an excellent region to conduct this pilot study. The study was conducted in an underserved community in San Pedro Sula, Honduras. The neighborhood was 31 hectares and was primarily composed of residential properties, although there were some small commercial stores including restaurants in the area as well. This community was selected due to a high incidence of dengue cases originating in this area and historic problems with container-inhabiting mosquitoes, which are known vectors of DENV, CHIKV, and ZIKV. Additionally, due to the lack of organized and consistent mosquito control, the need to implement alternative methods of controlling container mosquitoes was evident in this neighborhood.

### 2.2. Study Team and Training

A two-week workshop on basic mosquito biology, ecology, and control was delivered at Universidad Nacional Autónoma de Honduras (UNAH) in San Pedro Sula in December of 2018. The workshop attendees represented community leaders as well as other stakeholders including university faculty and students, doctors, nurses, engineers, and government officials. Topics included within the course were basic mosquito biology including the life cycle and blood-feeding behavior, mosquito identification, vector competency for arboviruses, and an overview of mosquito control using an integrated pest management technique. This information was delivered first in a lecture and reinforced through hands-on activities (rearing, trapping, and collecting mosquitoes; curation of insect specimens; use of insecticides and repellants; monitoring for insecticide resistance; use of insect growth regulators for mosquito control; use of a rapid diagnostic test for Zika; and community outreach). As part of the course, participants were trained on how to inspect residential and commercial properties for larval development sites and communicate with residents about these sites, their impact on human and animal health, and how to prevent those sites from producing mosquito larvae through source reduction. Workshop attendees were then able to apply this knowledge to home visits around their community.

The workshop was designed and taught by a Ph.D. student and university associate professor both with research focuses on mosquito biology, control, and/or mosquito/arbovirus interaction. There were no exclusion criteria imposed on workshop participants and anyone with an interest in attending was allowed to participate. Therefore, a range of education, background, and experience was represented among workshop participants.

Participant baseline knowledge of mosquito biology, control, and mosquito-borne diseases was assessed pre-training and post-training using a multiple-choice survey written in Spanish. Only participants that completed both pre- and post-training surveys were included in the analysis. Surveys were independent and based on participant efforts with no access to outside information. Scores were expressed as a percentage of the total questions answered correctly.

### 2.3. Survey and Community Visits

A survey was created that collected information on the number and type of containers located on the property as well as information on whether the individual had heard of Zika, dengue, or chikungunya. Additionally, information on the home construction, source of water, education level and number of people in the home, employment status, and vaccination status were collected. The study site was divided into subsections and each subsection was assigned to a team (2–3 individuals/team). That team was responsible for visiting all residences within their subsection.

Workshop participants were only allowed to participate in community visits after the completion of the workshop. Community visits were conducted the day following the completion of the workshop. During the visits, the study team first obtained verbal consent from the resident to inspect their property and provide information about mosquitoes. If the homeowner agreed, the survey questions described above were read aloud to the participant and the study team recorded their answers. Following the questionnaire, teams performed a property inspection where mosquito larval habitats were identified and quantified. Data on the size of the container was not collected. The variables of interest were (1) information on socioeconomic and other questions and (2) the prevalence and type of containers on the property.

**Figure 1:**
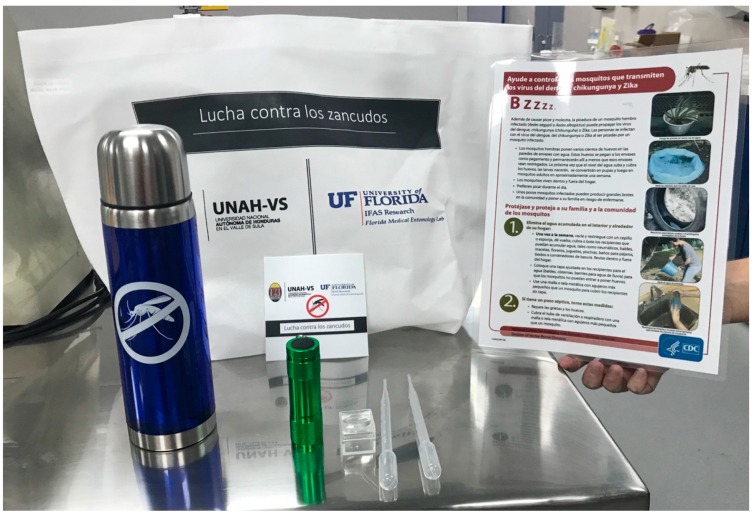
Materials provided to residents during community visit.

Containers were categorized as an air condition drip (puddle forming from the drip), bird bath, buckets, corrugated drainage pipes (water collects in recessed areas), flowerpots (including their base), garbage (any kind of small discarded/abandoned item), ornamental/decorative bowls or fountains, pet dishes, pools or boats (including covers over them that may be collecting water), rain gutters (especially those clogged with leaves), small ponds, tires, toys, trash bins (including both regular trash and recycling bins), water drainage, water storage containers (i.e., rain barrel, used by the homeowner to collect water for drinking/watering plants, etc.), water-holding plants (including bromeliads and other phytotelmata) or a wheelbarrow. Once the larval habitats had been identified, the study team provided information to the resident on how to prevent the containers from developing mosquito larvae. Informational materials and other supplies were left with the homeowner (Figure 1). This included an educational flyer (in Spanish) from the Centers for Disease Control and Prevention [32], a reusable tote bag with the slogan “Lucha contra los zancudos” (Fight against mosquitoes), an insulated stainless steel water bottle, pencils and stickers with the anti-mosquito slogan (as well as UNAH and University of Florida logos), a flashlight, magnifying glass, and plastic pipette for use in inspecting water-holding containers for mosquito larvae. These items were intended to reinforce the container elimination message even after the study team concluded their outreach. International Review Board (IRB) approval was obtained for this study (Protocol #IRB201802860).

### 2.4. Data Handling and Analysis

Descriptive statistics were used to summarize observed differences in the total number of containers on a property by different demographic and socioeconomic factors. Container type data did not fit a normal distribution (Shapiro–Wilk test) and were analyzed using a non-parametric analysis. To determine the most prevalent container type in the area, a Kruskal–Wallis test was used for analysis with container type as the independent variable. A paired sample *t*-test (one-tailed) was used to analyze surveys completed by workshop participants.

## 3. Results

Surveys were used to assess baseline knowledge about mosquito biology, control, and mosquito-borne diseases and to determine whether training improved knowledge. A paired sample *t*-test showed a significant, although modest, improvement in test scores following workshop training (*t = 5.02, df = 11, P = 0.0002*; mean ± standard deviation, pre-training, 65.14 ± 10.88%, post-training, 77.34 ± 11.29%).

**Figure 2:**
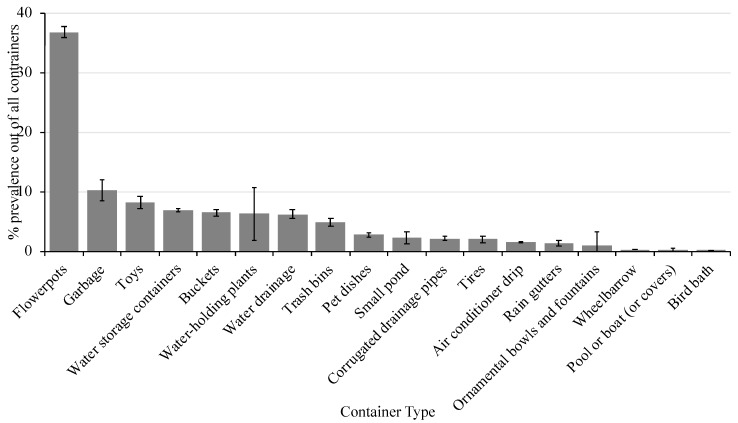
Percent prevalence of each container type (± SEM) in the study area.

A total of 105 surveys were completed (including property inspection) and educational materials were provided to the residents. During the property inspection, ~1500 containers were found to be holding water and there was an average of 15 (± 2.3) containers per residence. There was a significant effect on container type (*X*^2^ = 101.3, *P* ≤ 0.0001) with flower pots representing the most prevalent container type in the surveyed area (Figure 2). Flower pots made up 37% of the water-holding containers identified followed by garbage (10%), toys (8%), water storage containers (7%), buckets (6.5%), water-holding plants (6%), water drainage (6%), and trash bins (5%). The remaining container types each represented less than 3% of all containers identified in the area. The containers listed made up ~80% of all containers identified in the area and could be targeted in future campaigns.

Of the residents that were spoken to, 67% were female and the age of the individual spoken to ranged from 18 to 87 years. The majority of those surveyed lived in brick homes with zinc roofs and received their water regularly through the pipeline. Of those individuals that stored drinking water in containers, only 13% of them made sure to properly cover the containers to prevent oviposition by mosquitoes.

The average number of containers per residence did not vary greatly from the average (15 ± 2.3) between most socioeconomic factors or other survey questions, but some trends/variations were observed (Table 1). While most people in the community had heard of Zika, dengue, or chikungunya, 6% of those surveyed had not. The individuals that hadn’t heard of one of those mosquito-borne diseases had an average of nine more containers on their residence than individuals that had heard of these illnesses. Additionally, individuals living in homes without solid flooring had an average of nine more containers on their residence compared to those with a solid floor. Individuals with an incomplete primary education made up 22% of the population and had the highest number of containers per residence (21) compared to other individuals (11–15). 78% percent of those interviewed had children living in the home and 17% said that their children were not up to date on their vaccinations. At the residences where children were not up to date on vaccinations, there was an average of 17 more containers per residence compared to those residences where the children were up to date on vaccinations. Although we made no attempt to quantify the numbers of mosquitoes in each container habitat, the majority of the mosquitoes were identified as *Ae. aegypti*.

## 4. Discussion

The driving factors behind vector-borne disease transmission are not solely based on climate-related variables. Rather, risk of transmission is determined by a combination of social, ecological, and climatic conditions that influence the incidence of diseases such as dengue [33]. High human population density and a high prevalence of containers in an area create the ideal opportunity for dengue to spread among a population. Considering that *Ae. aegypti* and *Ae. albopictus* have a short flight range (maximum of 800 m, average of 200 m) [34,35], the burden on a specific community and neighborhood may be disproportionate to other neighboring areas. This characteristic emphasizes the importance of (1) engaging the community in vector control and container elimination and (2) developing highly targeted campaigns to maximize the impact of reducing container mosquito populations. Our survey of participants indicated an increase, although modest, in knowledge about mosquito biology, control, and mosquito-borne diseases. We caution against a strict interpretation of the participants score and overall knowledge of the topic given that only a single measurement was made using a single survey tool. Regardless, a change was observed in the anticipated direction.

A multi-country study in Asia showed that both knowledge and awareness of dengue and vector control activities had a significant impact on mosquito density in the area [33]. In the current study, despite the high dengue incidence in the study area, approximately 6% of those surveyed had never heard of Zika, dengue, or chikungunya. Interestingly, those individuals had an average of nine more containers (60% higher than the average) on their property than those that had heard of one of these diseases. These findings offer further support that knowledge of dengue can serve as a protective factor that may reduce risk of transmission. By educating residents about the risk associated with the presence of an abundance of water-holding containers, they may be more diligent about container elimination on their premises.

Effective mosquito control relies on the need to not only conduct larviciding and adulticiding, but also to identify and target the most productive containers in an area [33,36]. Similar findings were observed in a study conducted in eight countries. The authors of this study found that targeting only the most productive containers (approximately 50% of all containers) was equally as effective as targeting all types of containers, in reducing entomological indices for mosquitoes [37]. In another study reviewing the historical presence of *Ae. aegypti* in parts of Australia, regulation and community engagement in larval habitat removal were among the major reasons why *Ae. aegypti* and dengue were eliminated from the area [38]. These studies highlight the importance of community engagement and targeted elimination of types of water-holding containers responsible for higher production of invasive *Aedes* mosquitoes. The types of container present in an area varies greatly depending on geography and cultural practices [36,39,40,41]. Therefore, identifying the most prevalent and problematic containers in an area is a necessary step in controlling container mosquitoes. This approach allows for a maximum reduction in vector densities, and potential dengue incidence, in the area. While the present study did not monitor mosquito abundance, the addition of an autocidal gravid ovitrap in future studies would allow for both surveillance of the container mosquito populations and serve as a competitive oviposition source with the potential to aid in the control of these mosquitoes as well [42].

While the most prevalent container within this community was identified, this can vary greatly by the region the study is conducted in [43,44], the season in which the study is conducted [43,45], and even the way in which the container types are defined. Container mosquitoes demonstrate a large degree of flexibility in the type of container they will occupy based on availability. The present study was conducted during the rainy season, and therefore, outdoor containers that would not hold water long enough for a complete mosquito life cycle in the dry season were able to allow complete development. During the dry season, garbage may no longer act as a major container mosquito development site, and the utilized container may shift to containers that are consistently filled such as pet dishes or flower pot bases, a trend already observed in other studies [43,45]. Identifying changes in seasonality would require surveillance during both the wet and dry seasons and control measures should reflect this shift.

Similar to other studies, we found that certain social and economic factors were associated with the prevalence of containers on the property. Behaviors and factors associated with a higher number of containers on the property included a lack of solid flooring in the home, an incomplete primary education, storing drinking water in containers, and failing to have children up to date on vaccinations. While this does not represent a causal relationship, it supports the known positive association between poverty and vector-borne diseases. The storage of water in open containers as well as higher population density allow for greater risk of dengue transmission [46]. The prevalence of garbage within the study area could indicate that there is a lack of services to collect and dispose of the garbage [47], therefore preventing the elimination of one of the leading sources of habitats for container mosquitoes. In a study conducted on the West Nile virus incidence and socioeconomic factors, low income households, homes built before 1960, and the density of the housing were the most significant risk factors [48]. Older homes and low income were associated with a higher number of containers. Similarly, we observed in this area of San Pedro Sula that there was a high density of housing and the majority of these individuals were low income with higher numbers of containers on their premises. The lifestyle of individuals living in this area can also play a role in container elimination and therefore, dengue transmission. For example, living in homes without air conditioning, opening windows that do not have screens, inadequate disposal of garbage, and generally, not preventing items that can collect water from doing so all influence mosquito prevalence and their ability to feed on humans. These lifestyle behaviors could be due to a lack of knowledge related to mosquitoes and their development sites [49], a lack of resources [47], convention, or the inability to afford the items necessary for mosquito prevention (window screens, air conditioner, etc.).

A review of studies conducted in Brazil revealed that education and knowledge alone were not enough to influence the incidence of dengue [49]. Several studies conducted in Australia highlight similar points on the importance of not simply providing information to the community, but first aiming to understand the community’s beliefs and perceptions about the topic you are discussing [50,51,52]. The present study did not assess these factors, but as the program is expanded, incorporation of these considerations could enhance the sustainability of the program. Considering the community’s current knowledge and understanding, as well as the environment that has influenced that knowledge, allows for tailoring of the message at a community level [52]. The framework described here complements this engagement philosophy as we aimed to engage community leaders who had insight into community and other influences in the area. A strength of the present study lies in the fact that community members were not only provided educational material, but they were also trained on how to prevent specific containers around their home from harboring mosquitoes.

As the study conducted was a pilot study, there is significant room for improvement and limitations associated with the project. The short intervention time described here did not allow for monitoring behavior change within the community, the impact on the abundance of container mosquitoes and dengue transmission, etc. However, after the successful implementation of this campaign, future work on this topic can focus on increasing the intervention time and closely monitoring these outcomes. Additionally, workshop participants had varying levels of education, background, and experience, which could represent a bias. Individuals with a higher education or experience in a biological field may have been more receptive to the material presented.

The workshop curriculum presented was in depth and presented information that may not be directly applicable to community interventions like the intervention described here. The curriculum was also presented in English to a primarily Spanish-speaking audience (with translators). The workshop could be improved through (1) streamlining the curriculum and focusing on information that will aid in community engagement and (2) incorporating trainers who can deliver the workshop in the primary language of the audience.

In the future, the authors plan to expand this program to other regions that face significant challenges from container mosquitoes and associated diseases. Through improvements in the workshop and expansion of the program to other regions, it is possible that a training and certification program could be achieved. Such a certification would create a network of public health allies armed with the ability to address container mosquito control within their own communities. Empowering communities to lead this charge is critical to the sustainability of such a program.

Here, we demonstrated a method to train a diverse workforce of individuals to carry out container elimination campaigns in their local community. After the training, these community leaders and stakeholders were able to immediately apply that knowledge to a community and successfully identify the most prevalent water-holding containers in the area and trained the community on how to eliminate them. Applying this knowledge in a community that experiences a high incidence of dengue could have significant impacts on both the risk of endemic and epidemic transmission. Engaging stakeholders in these mosquito control activities is considered a crucial component of building relationships and support networks that facilitate an integrated vector management program [53]. However, this project was limited because it was a pilot study. Therefore, we only collected data from one community and trained one cohort of individuals. Future work could expand training to multiple groups in other areas of Honduras and collecting similar data from diverse communities, as well as the inclusion of a rapid and inexpensive multiplexed test for dengue, chikungunya, and Zika viruses in clinical and mosquito samples [54,55]. While the results of our study are specific to this community in San Pedro Sula, the methods can be generalized to target other communities and create highly precise information based on the community that is being targeted or the group that is being trained.

## 5. Conclusions

A two-week workshop in San Pedro Sula, Honduras was successful in training a diverse group of community leaders and other stakeholders on various aspects of mosquito biology and control. Workshop attendees were able to immediately apply this knowledge to an underserved community in the city with high dengue incidence. A survey was conducted that quantified the types of containers in the area and community members were trained on eliminating those containers. The most prevalent containers in the community were flowerpots, garbage, toys, water storage containers, buckets, water-holding plants, water drainage, and trash bins. Additionally, never hearing of Zika, dengue, or chikungunya, the lack of solid flooring in the home, drinking water stored in containers, having an incomplete primary education, and failure to have children in the home up to date on vaccinations were all associated with higher numbers of containers on the property. This study offers a framework for training community leaders and stakeholders in engaging their local communities in vector control and appropriately targeting the most numerous and problematic containers.

## Figures and Tables

**Table 1:** Average number of water-holding containers by survey question.

	n	% of Category	Average # of Water-Holding Containers on Property
Total	105	100	15
**Have you heard of Zika, dengue, or chikungunya?**			
Yes	99	94.3	14
No	6	5.7	23
**What is the floor material?**			
Cement	50	47.6	16
Ceramic	29	27.6	12
Mosaic	20	19.0	15
Soil	5	4.8	23
Missing	1	1.0	7
**Is drinking water stored in containers?**			
Yes	39	37.1	19
No	66	62.9	12
**What is your education level?**			
None	1	1.0	4
Incomplete Primary	23	21.9	21
Complete Primary	24	22.9	11
Incomplete High School	13	12.4	15
Complete High School	24	22.9	14
Incomplete University	9	8.6	12
Complete University	11	10.5	14
**Are children in the home up to date on vaccinations?**			
Yes	68	82.9	12
No	14	17.1	29
